# Should hydrogen therapy be included in a musculoskeletal medicine routine?

**DOI:** 10.12688/f1000research.9758.1

**Published:** 2016-11-10

**Authors:** Sergej M. Ostojic

**Affiliations:** 1 Faculty of Sport and PE, University of Novi Sad, Novi Sad, Serbia; 2 University of Belgrade School of Medicine, Belgrade, Serbia

**Keywords:** FDA, Molecular hydrogen, Rheumatoid arthritis, Soft tissue injury

## Abstract

Molecular hydrogen (H _2_) has recently been recognized as a potential novel therapeutic agent in biomedicine. Initially proposed to be a possible treatment for certain types of neuromuscular disorders, cardio-metabolic diseases and cancer, H _2_ improved clinical end-points and surrogate markers in several clinical trials, mainly acting as an anti-inflammatory agent and powerful antioxidant. In this paper, the medicinal properties of H _2_ in musculoskeletal medicine are discussed with the aim to provide an updated and practical overview for health professionals working in this field.

## Background

As the oldest and the most abundant molecule in the universe, molecular hydrogen (H _2_) has been traditionally recognized as a biologically inert gas. However, several trials in the past 10 years reported beneficial effects of H _2_ in the clinical environment, revealing its possible role as a novel therapeutic agent in medicine ^1– 5^. Usually administered orally or via inhalation, H _2_ improves both patient- and clinician-reported outcomes, and biomarkers of different pathologies and disorders, from metabolic diseases to chronic systemic inflammatory disorders to cancer [for detailed review see Ref. 6]. Its clinical relevance seems to be particularly notable in the musculoskeletal medicine, with several small-scale short-term studies ^7– 9^ reporting that H _2_ was able to restore the health and functional abilities of patients after acute injuries or chronic illnesses affecting the muscles and bones. Since musculoskeletal conditions account for a large proportion of a general practitioner's workload ^10^, one might consider H _2_ as a promising medication or adjuvant that could alleviate these prevalent conditions. In this opinion paper, the medicinal properties of H _2_ in musculoskeletal medicine are discussed to provide an updated and practical overview for health professionals working in this field.

## Promising results from preliminary studies

Being prompted by the prominent effects of H _2_ on disuse muscle atrophy, cartilage trauma, and osteopenia in animal studies ^11– 13^, a number of clinical investigators from 2010 onwards evaluated the effectiveness of H _2_ in patients suffering from different muscle and bone ailments – from sprains and strains to chronic joint disorders to myopathies ^7– 9^. Typically, these studies were designed as single-blind pilot trials, with small sample sizes (< 40 participants) and of short duration (≤ 12 weeks). Although limited in size and scope, those studies can provide early support for specific therapeutic claims about H _2_ in musculoskeletal medicine. In a first trial, a combination of oral and topical H _2_ resulted in a faster return to normal joint flexibility in 36 young men who had suffered sports-related soft tissue injuries, when administered for 14 days as a complementary treatment to a traditional medical protocol for soft tissue injuries ^7^. H _2_ intervention (hydrogen-rich packs 6 times per day for 20 min and 2 g of oral H _2_ daily) was found to augment plasma viscosity decrease after an injury, while other biomarkers of inflammation (C-reactive protein, interleukin-6) and clinical outcomes (pain scores at rest and at walking, degree of limb swelling) were not affected by the intervention ^7^. Another study in Japan reported that drinking 530 ml of a liquid containing 4 to 5 ppm of H _2_ every day for 4 weeks significantly reduced disease activity in 20 patients with rheumatoid arthritis, as evaluated by changes in the degree of tenderness and swelling in 28 joints and C-reactive protein levels ^8^. H _2_ was administered as an adjuvant to regular disease-modifying anti-rheumatic drugs and biological drugs, with the efficacy of H _2_ found to be not inferior comparing to abatacept, methotrexate or a combination of two. In total, 47.4% of patients went into remission, with anti-citrullinated protein antibody (ACPA)-positive patients (ACPA levels above 300 U/mL; patients with worse prognosis and higher rates of erosive damage) responding best to the treatment. Finally, the consumption of water containing a high concentration of H _2_ (31% saturation) for up to 12 weeks improved surrogate markers of muscle pain and fatigability in 22 patients with inherited and acquired myopathies treated with low-dose prednisone ^9^. Taken together, the above studies seem to pave the way for a future use of H _2_ therapy in musculoskeletal medicine.

## Take it with a grain of salt

Compared with conventional treatment protocols in musculoskeletal medicine, based on drugs and methods that are well-described with respect to efficacy and safety ^14, 15^, H _2_ still has a long journey ahead before it can be recognized as a common remedy in this medical discipline. At the moment, H _2_ therapy is not adequately described in terms of approval, labeling, side effects, and pharmacovigilance information in musculoskeletal medicine. There are no dose escalation studies yet, and the optimal and safest dose range for H _2_ remains unknown; furthermore, no federal agency or industrial entity provides appropriate patient counseling information about H _2_. The US Food and Drug Administration (FDA) recently issued a notice (GRAS Notice No. 520) ^16^ of a claim that the use of H _2_ solubilized in water (up to a concentration of 2.14%) is generally recognized as safe (GRAS) when it is added to beverages and beverage containers in order to prevent oxidation. Based on the information provided by the H _2_ gas-manufacturing company, as well as other information available to the FDA, the agency had no questions about the conclusion that hydrogen gas is GRAS under the intended conditions of use. However, the FDA has not made its own determination regarding the GRAS status of the subject use of H _2_ gas ^16^. This seems to be the only formal information currently available concerning the use of H _2_ in food or medicine! Despite this lack of formal approval, there are many formulations and devices widely available in the market that claim to supply H _2_ for the use in musculoskeletal disorders, from gas-producing machines to dietary supplements and beverages, with H _2_ amount varying greatly across the different products. Consequently, consumers might be exposed to easy-to-acquire but questionable products containing H _2_.

Among other important medical issues that need to be addressed, including long-term safety or pharmacokinetics, the main question remains whether H _2_ should be considered as a dietary supplement or a medicine, since the FDA declares that a product intended for inhalation (such as H _2_) is not a dietary supplement ^17^. Therefore, considering H _2_, or at least some H _2_ forms, for much stricter assessment and regulation by formally recognizing it as a drug in the future, might be more appropriate for this promising bioactive gas. So, it will take many more studies and tighter regulation before H _2_ therapy can be endorsed as a routine protocol (or adjuvant to standard treatment) in musculoskeletal medicine. In the meantime, H _2_ should be regarded as an experimental agent and not recommended to treat muscle or bone conditions in the general population.
