# Supplementary material for: Should hydrogen therapy be included in a musculoskeletal medicine routine?
Source: F1000Res. 2016 Nov 10;5:2659. [Version 1] doi: 10.12688/f1000research.9758.1 (PMC5147523; doi:10.12688/f1000research.9758.1)
Supplement: Peer review history

# Peer review: Referee response for version 1

**DOI:** 10.5256/f1000research.10520.r17912

**Published:** 2016-12-08

**Authors:** Steven R. Brenner

**Affiliations:** 1 Department of Neurology and Psychiatry, Saint Louis University School of Medicine, Saint Louis, MO, USA

The title is appropriate with reference to the content of the article.

 The article is a review of the literature with reference to utilizing molecular hydrogen to enhance sports related injuries.

 After a detailed review of the literature, the conclusion is there is not enough information to make any solid recommendation concerning utilizing molecular hydrogen to treat sports related injuries, so the implication is probably molecular hydrogen doesn't improve recovery from sports related injuries enough to make any difference.

 This appears to be a good review of the related literature.

I have read this submission. I believe that I have an appropriate level of expertise to confirm that it is of an acceptable scientific standard.

# Peer review: Referee response for version 1

**DOI:** 10.5256/f1000research.10520.r18096

**Published:** 2016-12-08

**Authors:** Xiaoli Sun, Ning Zhang

**Affiliations:** 1 Department of Diving Medicine, Second Military Medical University, Shanghai, China; 2 Department of Naval Aeromedicine, Second Military Medical University, Shanghai, China

This opinion paper provides an undated and practical overview on the properties of molecular hydrogen in musculoskeletal medicine. The paper focuses on the preliminary studies of H2 on musculoskeletal medicine, and the concerns over the general use of products containing H2. I sympathize the author’s prudent attitudes, which toward the hydrogen should be regarded as an experimental agent and not recommended to general use provisionally. However, I think this paper should also mention the long-term diving practices which high pressure hydrogen inhalation involved to prove the possible safe use of H2 gas.

We have read this submission. We believe that we have an appropriate level of expertise to confirm that it is of an acceptable scientific standard.
